# Improvement of YX42° Cut LiTaO_3_ SAW Filters with Optical Proximity Effect Correction Method

**DOI:** 10.3390/mi14010205

**Published:** 2023-01-13

**Authors:** Ping Luo, Yali Zou, Xinyu Yang, Juntao Li, Xuan Huang, Jian Zhou, Xing Han, Yushuai Liu, Yuhao Liu, Tao Wu

**Affiliations:** 1 School of Microelectronics, Shanghai University, Shanghai 200444, China; 2 Shanghai Bawnovation Co., Ltd., Shanghai 200120, China; 3 School of Information Science and Technology, ShanghaiTech University, Shanghai 201210, China; 4 Shanghai Institute of Microsystem and Information Technology, Chinese Academy of Sciences, Shanghai 200050, China; 5 University of Chinese Academy of Sciences, Beijing 100864, China; 6 Shanghai Engineering Research Center of Energy Efficient and Custom AI IC, Shanghai 201210, China

**Keywords:** surface acoustic wave (SAW) filter, optical proximity effect (OPE), optical proximity effect correction (OPC)

## Abstract

Due to the influence of the optical proximity effect (OPE), it is easy for a pattern of photoresistance to be inconsistent with a design pattern, thus damaging the performance of a SAW resonator. To solve this problem, this paper proposes an optimization method for SAW filters based on optical proximity correction (OPC). This method can avoid the tip discharge problem of SAW filters by suppressing the problem of rounding and shrinking of dummy electrode and electrode tail caused by OPE. This method increases the quality factor (Q) of the SAW resonator and thus decreases the insertion loss of the SAW filter. The filter increases the bandwidth by 1.8 MHz at −1.5 dB after applying the OPC method. Additionally, it improves the stability of the filter under high power conditions.

## 1. Introduction

Surface Acoustic Wave (SAW) is widely used in SAW filters and SAW sensors. Among them, the SAW sensor is divided into delay line type and resonator type, which is a kind of sensor using a SAW device as a sensing carrier. It has the advantages of high sensitivity, low cost, low power consumption, miniaturization and direct frequency signal output. With the increasing demand for miniaturized, low power consumption and highly integrated mobile devices, the Surface Acoustic Wave (SAW) filters are widely used [1].In recent years, there have been many commercial SAW filters on the market, e.g., (1) 42° YX Cut LiTaO_3_ SAW [2]; (2) Incredible High-Performance SAW (I.H.P SAW) [3,4,5,6,7]; (3) 128° YX Cut LiNbO_3_ Temperature Compensate SAW [8]. The I.H.P. SAW introduces a functional layer and a high acoustic velocity layer under the piezoelectric crystal layer. The active layer is usually used for frequency compensation; the high-velocity layer is used to limit energy propagation and increase the speed of the acoustic surface waves [3,4,5,6,7]. Temperature Compensate SAW(TC-SAW) has a complementary temperature layer-SiO_2_, which can play a role in temperature compensation and improve the Q value of the SAW resonators. However, the I.H.P SAW and TC-SAW both have the problem of high sub-mode transverse wave response [3,4,5,6,7,8].

Compared with I.H.P SAW and TC-SAW, 42° YX Cut LiTaO_3_ SAW filter does not have a high sub-mode transverse mode response which means it does not require complex interdigital transducer (IDT) structures and more process steps. The process steps for fabricating SAW filters are fewer, so the process is simpler and less costly. When designing SAW filters, the problem of transverse SAW leakage can be avoided by adding dummy electrodes on the interdigital transducer (IDT). The dummy finger between the electrode and the bus bar creates an electrode gap. Although a narrow electrode gap (a narrow gap means that it is close to the light source wavelength of the lithograph or less than it) can help suppress the lateral leakage of SAW [2,9,10], it can also cause significant OPE during the lithography process [11,12,13]. Hence, for SAW resonators, OPE will cause edge rounding and inward shrinkage of the electrodes and dummy electrodes on both sides of the electrode gaps, which can reduce the effectiveness of transverse SAW leakage suppression. In addition, edge rounding may result in tip discharges [14]. It is generated because the alternating voltage acting on the narrow electrode gap excites a large electric field that ionizes and breaks through the air between the gaps. This process may occur more easily, especially when the conductor’s face is convex. As a result, the edge rounding can short-circuit the dummy electrodes and the electrodes, affecting the power handling of the SAW filter.

## 2. SAW Resonator

**Figure 1:**
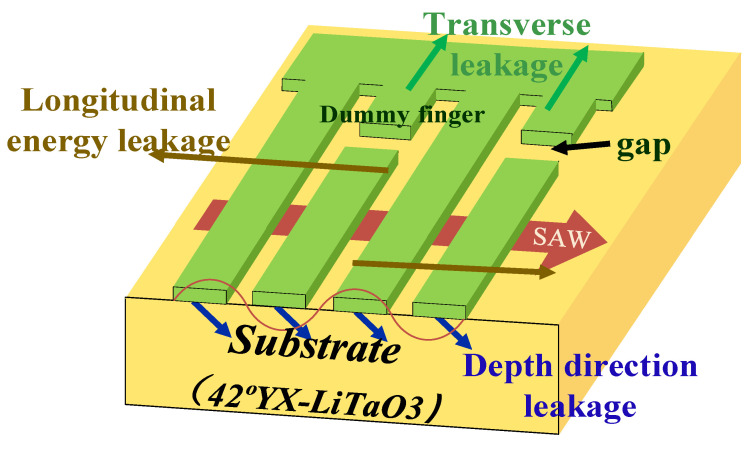
Energy leakage chart.

There are three main types of SAW energy leakage, divided into three directions: longitudinal, transverse, and depth [15], as shown in Figure 1. For the longitudinal energy leakage, it is usually suppressed by using reflective grids. Since it is difficult to suppress the energy leakage in the depth direction on a single-layer substrate, this paper does not consider how to reduce it. It is generally helpful to introduce an appropriate length of virtual electrodes into the resonator and retain a proper electrode gap to suppress the lateral leakage of the SAW and to improve the quality factor of the SAW resonator, which will be verified by simulation results later in this section.

When designing SAW filters with a high center frequency, the OPE will be evident because the width of the electrode gaps, electrodes, and virtual electrodes are close to the wavelength of the light source of the lithograph. In this case, the lithograph can position itself to the optimal lithographic conditions by adjusting the light’s exposure energy and focal length, but cannot compensate for the deviations caused by the OPE in both X and Y directions simultaneously. It is assumed that the width direction of IDT is X direction and the length direction of IDT is Y direction. Therefore, when the lithograph ensures that the width of the IDT is consistent with the design pattern, the virtual electrodes and electrodes on both sides of the electrode gap tend to tail-end rounding. Additionally, it is accompanied by inward shrinkage. All these changes weaken the effect of suppressing lateral SAW leakage. The expression of quality factor (*Q*) is shown in Equation (1) [16]. When the SAW energy leakage becomes large, it corresponds to a decreasing in the stored energy and an increasing in the consumed energy per cycle. It can be seen from Equation (1) that the *Q* value decreases. Therefore, the rounding and inward contraction of the virtual electrodes and electrode tails reduce *Q* of the SAW resonator.

(1) $$Q=2\pi \frac{Stored\;peak\;energy}{Energy\;consumption\;per\;cycle}$$

### 2.1. Simulation

**Figure 2:**
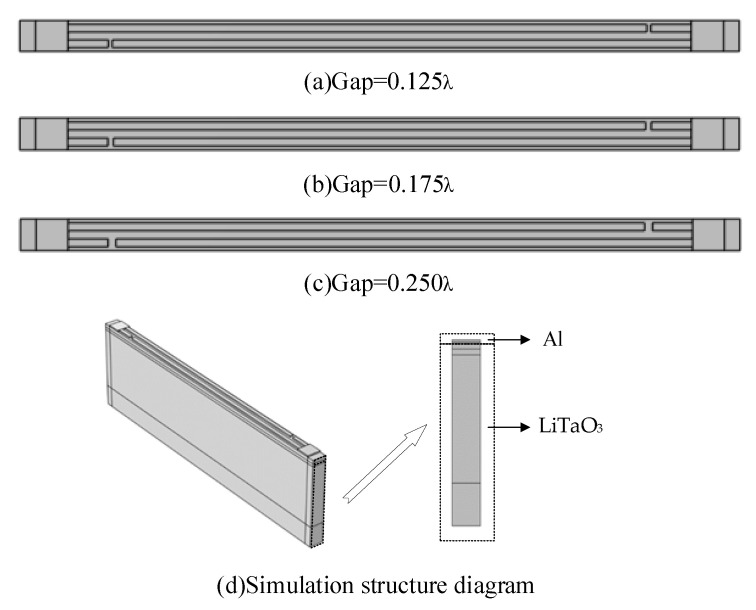
Diagram based on the simulation parameters in Table 1.

To verify the previous statements and explore the effects of virtual electrodes, the length of the electrode gap and OPE on the resonator performance, the finite element analysis method (FEM) is applied to simulate and analyze. The parameters are shown in Table 1 [17]. In order to reduce the computational effort of the simulation while ensuring the accuracy of the simulation, the simulation uses a pair of IDTs to analyze the mode of the 42° YX Cut LiTaO_3_ single-port resonator using periodic boundary conditions. The three-dimensional geometric model of the single-port resonator is established with the electrode parameters shown in Table 1 and the simulation model shown in Figure 2.

### 2.2. Analysis of Simulation Results

**Figure 3:**
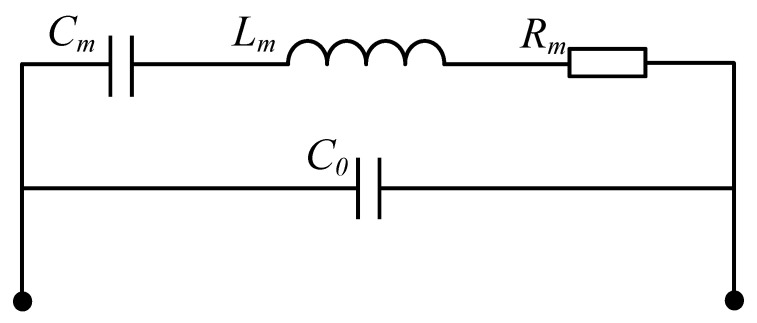
BVD model of a piezoelectric resonator.

The piezoelectric resonator is represented by the Butterworth-Van Dyke model (BVD), BVD is a circuit model based on electrical parameters, as shown in the Figure 3 where $C_{0}$ is the static capacitance, $C_{m}$ is the mechanically related dynamic capacitance, $L_{m}$ is the mechanically related dynamic inductance, and $R_{m}$ is the mechanically related dynamic resistance. The mechanical/elastic losses (including energy leakage of the system, etc.) are included in Rm. From Figure 3, it can be seen that the input impedance can be expressed as:

(2) $$Z=\frac{1}{jwC_{0}+1/\left(\frac{1}{jwC_{m}}+jwL_{m}+R_{m}\right)}$$

**Figure 4:**
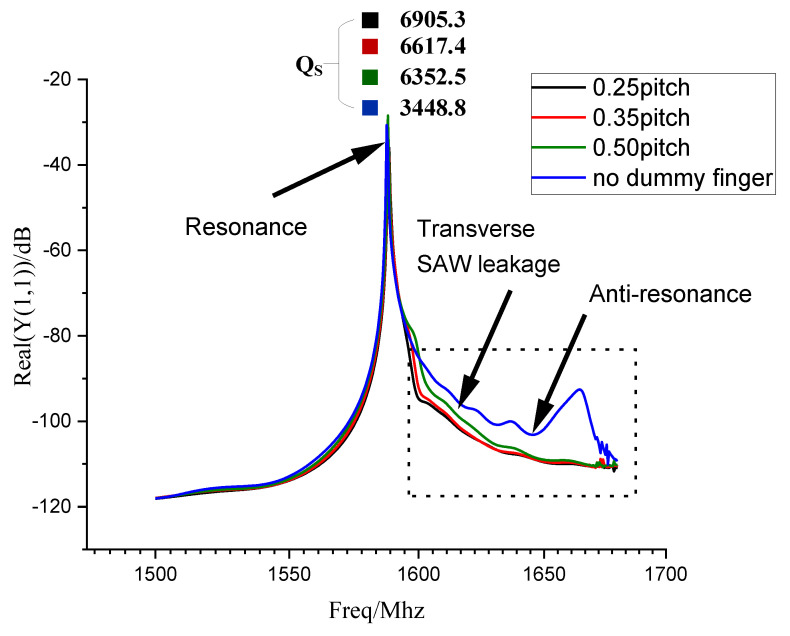
Comparison of conductance with and without dummy electrodes. First, two cases were simulated: one without virtual electrodes and the other with virtual electrodes. Then, three gaps were simulated in the case of virtual electrodes: 25% pitch, 35% pitch and 50% pitch. In the dotted black box, if the curve’s position is lower, the degree of lateral SAW leakage is more minor.

The input admittance is inversely proportional to the input impedance. From Equation (2), when $R_{m}$ becomes larger, the input impedance (Z) will become larger, and the input admittance (Y=1/Z) will become smaller. Therefore, the dissipation will be smaller when the conductance between resonant frequency and anti-resonant frequency is more minor. However, the change in the simulation group of Figure 4 is only the electrode gap, so the difference in the conductance is only related to the leakage loss. From the above analysis, it is clear that for the control group in Figure 4, the lower the admittance curve is, the less the transverse SAW leakage will be.

As shown in Figure 4, comparing the simulation results of 50% pitch and no virtual electrode, it shows that the Q value of the former resonant frequency is larger than the latter. In the black dashed box in Figure 4, the conductivity curve with the virtual electrode is significantly lower than without the virtual electrode, which indicates that the resonator impedance is significantly increased by the virtual electrode. Thus, in terms of the resonator, the lateral SAW leakage with dummy electrodes is more minor than that without dummy electrodes. So, the dummy electrodes can effectively suppress the lateral SAW leakage. Comparing three different electrode gaps: 25% pitch, 35% pitch, and 50% pitch, when the electrode gap is small, the position of the conductivity curve will be lower, as shown in the dotted black box in Figure 4. It means that the suppression of lateral SAW leakage gets better as the electrode gap decreases. From Equation (1), it is evident that the Q value of the resonator increases as the electrode gap decreases. OPE will cause the electrodes to shrink, which means the electrode gap will become larger. Therefore, OPE will reduce the quality factor of the resonator.

**Figure 5:**
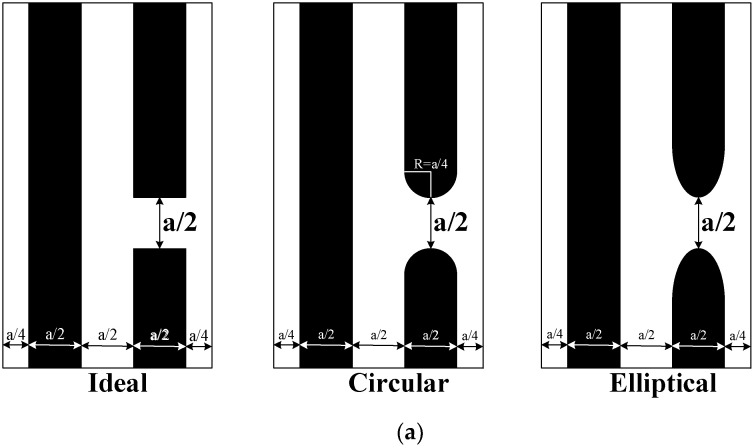

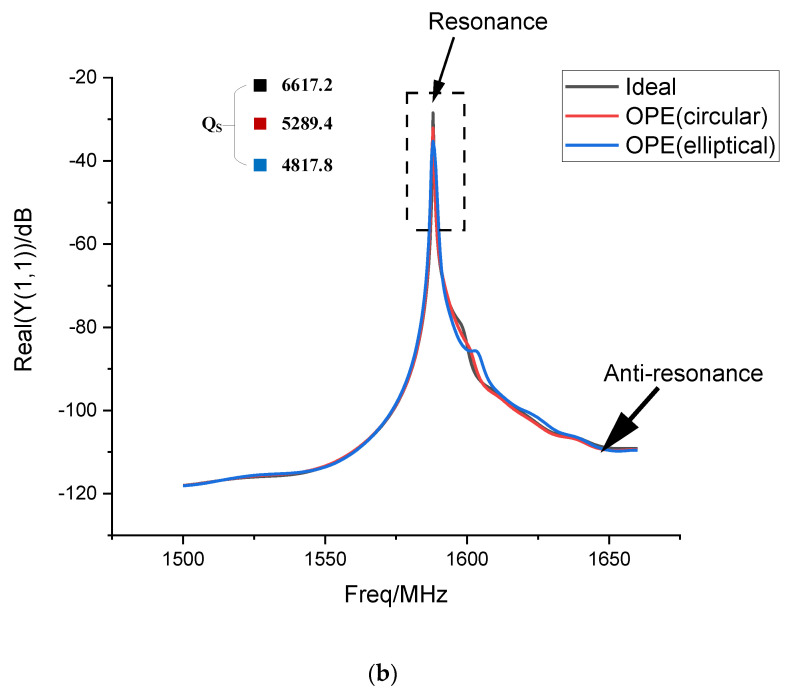
Effect of tail-end graphic distortion at the same gap size: (**a**) ideal pattern, circular pattern, elliptical pattern, (a = pitch); (**b**) simulation results based on (**a**). The dotted black box is a local magnification of the resonance frequency.

According to the simulation results above, the electrode gap effectively suppresses the lateral SAW leakage. Next, we will simulate and analyze the extent of deformation at the end of the electrode gap. In Figure 5a, the tail-end cases of the three electrodes are drawn. To analyze qualitatively, we maintain a constant electrode gap, which is 50% of the total pitch.

Figure 5b shows simulation results for different electrode tail-end deformations. The difference between the three cases is not evident in terms of the overall trend of the conductivity curves. As far as the Qs of the resonant peak are concerned, the Qs will decrease as the electrode tail’s extent of deformation increases. Therefore, the deformation caused by the OPE will reduce the Q value of the resonator.

In Figure 4 and Figure 5, we simulated the IDT regarding the electrode gap and the extent of tail-end deformation. They correspond to the two effects of the OPE on the ends of the electrode gap inward shrinkage and tail-end rounding, respectively. It can be found that the OPE is destructive to the resonator’s performance, which will reduce the Q value of the resonator. According to the simulation results in Figure 4, a narrow virtual electrode gap can suppress the SAW lateral leakage and thus improve the Q of the resonator. Since the OPE increases the electrode gap, the OPE will destroy the resonator performance and reduce the Q value. On the other hand, the simulation results in Figure 5 show that the electrode tail deformation will reduce the Q value of the resonant frequency of the resonator, and the OPE will cause the electrode tail deformation. Overall, the OPE has a negative impact on the Q value of the resonator in terms of both increasing the electrode gap and causing the electrode tail deformation.

As mentioned earlier, the OPE will round the electrodes at both ends of the electrode gap, which will affect the power handling capability of the resonator.

The equation for the electric field *E* near the conductor surface versus the charge density is shown in Equation (3), where σ is the charge surface density and ϵ is the radius of curvature.

(3) E=σ/ϵ

If the area of the trailing end is reduced under the influence of other factors (e.g., OPE), the radius of curvature will be smaller at the end of electrodes and dummy electrodes. The radius of curvature is more significant; the charge surface density will be greater in insulated conductors. Therefore, it can be seen from Equation (3) that when the charge remains constant, the charge density increases due to the area becoming smaller, which eventually causes the electric field to increase. At the same time, the forceful alternating electric field tends to excite the tip discharge, which ionizes the air. It will cause the conductivity of the electrodes and virtual electrodes, which ultimately disrupts the regular operation of the SAW resonator.

In summary, we know that the OPE has three effects on the SAW resonator.

1. Distortion of pattern edges occurs in high-frequency parts such as edges and corners, which will reduce the Q value and affect the performance of resistors.

2. The electrode gap will increase as the electrodes at the ends of the electrode gap shrink inward. It will weaken the suppression of lateral SAW leakage and reduce the Q value of the resonator.

3. Rounding of the electrode tails tends to induce tip discharge, affecting the filter’s power handling capability.

## 3. Optical Proximity Effect Correction (OPC)

### 3.1. Optical Proximity Effect (OPE)

Photolithography is an essential part of the filter manufacturing process, an optical imaging process that allows the design pattern to be transferred to the photoresist through the photomask. However, OPE issues in the actual photolithography process will have an impact on the image quality. OPE is caused by diffraction and light interference in the imaging process [11]. Among them, diffraction is universal, and only when the wavelength of the light source is larger than the diameter of the obstacle or small hole that the diffraction effect is significant [18]. Therefore, the optical proximity effect becomes more pronounced when the size of the designed pattern is more petite.

**Figure 6:**
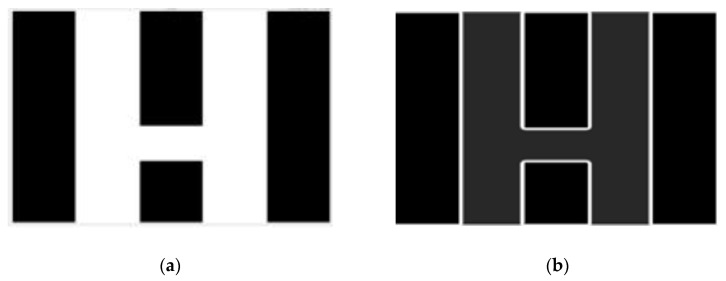
High-pass filtering of IDT local images with high-pass filtering: (**a**) partial diagram of IDT; (**b**) after filtering the graph by Gaussian high-pass filter.

**Figure 7:**
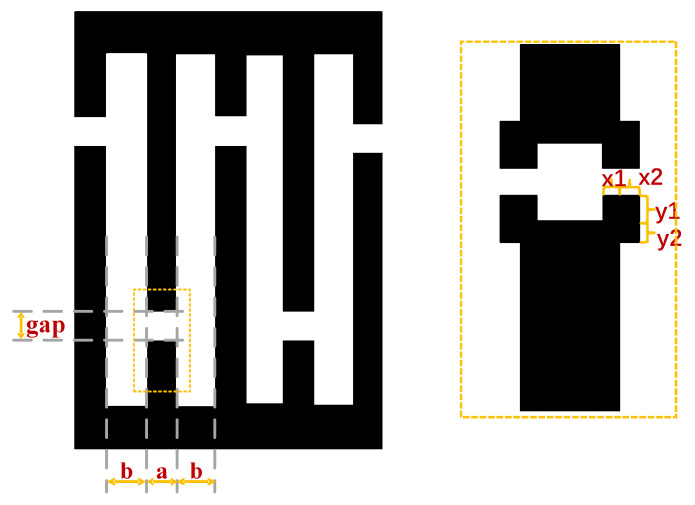
OPC compensation for adding patterns to photomasks.

The photolithographic imaging system is similar to a partially coherent nonlinear low-pass filter system, which reduces the energy of the high-frequency component of the image during imaging and allows the low-frequency component of the image to pass through [19]. This phenomenon will lead to weak light intensity at the edges and corners, making it difficult to image this part. As a result, OPE generally results in rounding corners, narrowing lines, shortening line ends, etc. In Figure 6, the image is processed through a Gaussian high-pass filter to explain why the missing edges and corners are missing when the SAW resonator is fabricated. Figure 6a shows the local image of the IDT, and the white part in Figure 6b is the image of the high-frequency part after high-pass filtering. Thus, the edges and corners are the high-frequency part of the graph. Similarly, with the nonlinear low-pass filter of the lithography imaging system [19], the lithography pattern of the SAW filter also shows missing edges and corners. We can pre-process the photomask to compensate for the part of the SAW resonator that tends to be lost. It can offset the image loss caused by optical proximity effects. The lithograph can control the light source to achieve the desired photoresist pattern by adjusting the focus and exposure energy; however, the lithograph cannot ensure that the lateral and vertical dimensions of the photoresist pattern are consistent with the design simultaneously. The photolithography can be adjusted to itself to compensate for the accuracy of the pattern in the vertical direction. For the others, we use external additive patterns to achieve as in Figure 7. Therefore, we apply OPC to the SAW filter design. The principle is that the OPE sacrifices the added pattern to obtain the desired pattern in the design. Image resolution can be improved using optical proximity effect correction [13]. By changing the image of the photomask, the pattern on the photoresist can be aligned with the design.

### 3.2. Application of Optical Proximity Effect Correction to SAW Filters

The SAW resonator is composed of IDTs and reflection gratings. When the SAW filter is applied to the high-frequency band, the line width of the resonator approaches the wavelength of the lithography light source. The optical proximity effect will significantly affect the lithography process, and it is challenging to ensure that the photoresist pattern in the critical areas (dummy fingers, electrodes, electrode gaps) is consistent with the design pattern. This will diminish the SAW resonator’s performance and trigger the electrode’s tip discharge at both ends of the electrode gap.

Therefore, it is necessary to apply optical proximity effect correction to the local position of the IDT to improve the performance and stability of the SAW filter.

There are two methods used for the correction of OPE. One is the rule-based empirical method, and the other is the model method based on the photolithographic model [11,20,21,22,23,24,25]. For the SAW filter, there are a few patterns to be corrected, the electrode tails at the ends of the gap and the virtual electrode tails. Therefore, this paper uses a rule-based empirical approach to fix the SAW filter. Therefore, it is necessary to develop correction rules.

In Figure 7, ‘a’ is the finger’s width, ‘b’ is the finger spacing, and ‘gap’ is the width of the gap between the virtual electrode and the electrode. The graph for correction is a rectangle, where x1, x2, y1, and y2 are the parameters of the rectangle. However, contrary to the traditional list-based rule method, this experiment will establish a linear relationship between the parameters of the compensation graph and the design parameters. There will be different linear relationships at different wavelength ranges. The relations are x1 = k × a, x2 = k × b, y1 = k × gap, y2 = x1 × y1/x2, and k belongs to [0.1, 0.15, 0.2, 0.25, 0.3]. Finding the optimal co-efficient k for each wavelength value is crucial to determining the design rule for optimal compensation. Experimental results for finding the optimal k are presented in the next section.

## 4. Experimental Results and Analysis

**Figure 8:**
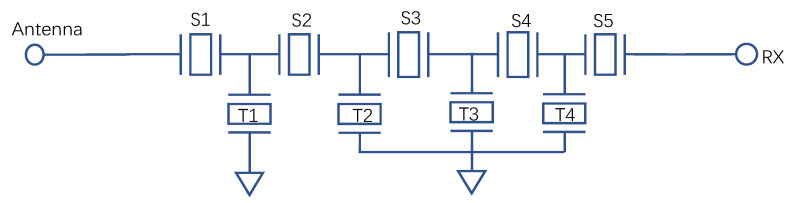
Topology of a SAW filter.

This paper uses a ladder SAW filter as an example for the experiment. The circuit structure of this filter consists of five stages. In Figure 8, S1–S5 are series resonators, T1–T4 are parallel resonators, and T1, T2–T4 are connected to the ground. The electrical signal is input from the antenna port and then passed through the electrical−acoustic−electrical conversion of each resonator to the RX port to output the electrical signal.

The following is an example of a series and a parallel resonator to analyze the combined position of the ladder filter and the effect of the resonator’s Q value on the filter’s insertion loss.

**Figure 9:**
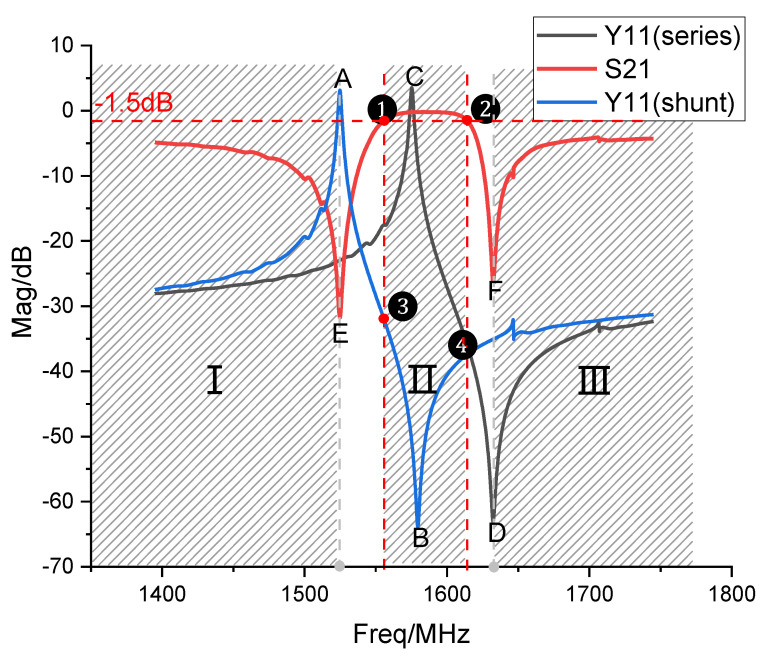
Ladder filter consisting of one series and one parallel resonator.

In Figure 9, Y11(series) is the input admittance curve of the series resonator, Y11(shunt) is that of the parallel resonator, and S_21_ is the insertion loss curve of the filter composed of one series and one parallel resonator. The shaded parts I and III are the resistive bands. An auxiliary line of −1.5 dB is made for the insertion loss curve of the filter, intersecting at ➀,➁. The shaded part II is the passband, and the blank region is the transition band. Figure 9 shows that the frequencies of A and E are the same, and those of F and D are the same. It indicates that the frequencies of the parallel resonator’s resonant frequency and the series resonator’s anti-resonant frequency determine the frequency locations of the filter’s out−of−band rejection points E and F, respectively. The anti−resonant peaks of the parallel resonator and the anti-resonant frequencies of the series resonator are both located in−band, so they define the width of the band.

However, if the Q value of the resonator is higher, the loss will be lower. In Figure 9, with the Q value at points A, B, C, and D increasing, the insertion loss S_21_ will be more stable. If the filter’s insertion loss is to be reduced, the resonator’s quality factor at the resonant and anti-resonant frequencies needs to be increased.

Based on the above circuit topology, the SAW filters with and without OPC were fabricated separately. Next, this chapter will compare and analyze the test results before and after the filter correction. The wavelength range of the filters for this experiment was 2.42 microns to 2.53 microns. The difference between the wavelength values of the parallel and series resonators is minimal. So, the wavelength of 2.42 microns was used as the reference for the rule experiment. The metal duty ratio is 0.5, and the electrode gap is 400 nanometers. A factor k will correspond to a resonator, so a wavelength corresponds to a corrected and uncorrected resonator.

**Figure 10:**
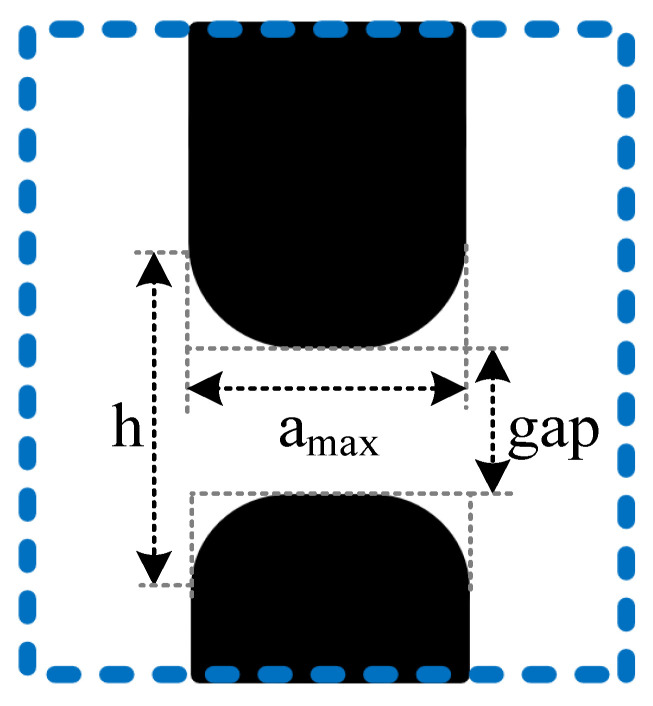
The parameters that SEM needs to measure.

The SEM maps are measured separately for each correction parameter; the parameters to be measured are shown in Figure 10.

Table 2 shows that when k = 0.3, the a_max_ is greater than the ideal value and is therefore discarded. Comparing the other correction schemes, it can be seen that the visual correction is best when k = 0.25.

**Figure 11:**
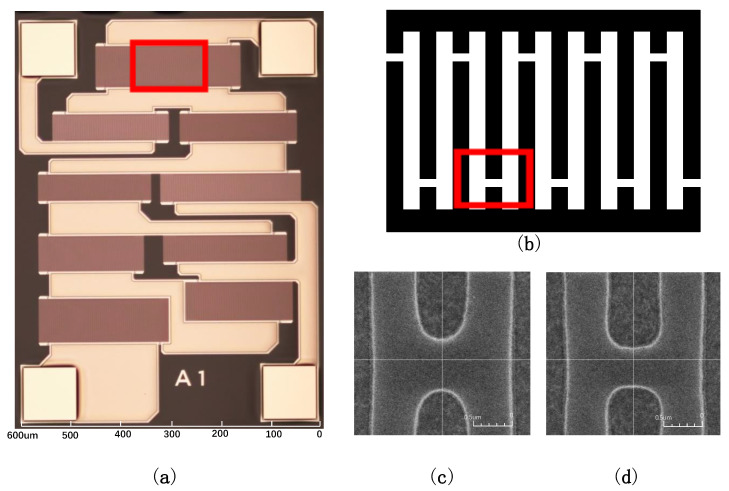
The physical map of the filter and local SEM comparison before and after OPC at both ends of the electrode gap: (**a**) the physical map of the filter; (**b**) the local schematic of the resonator in (**a**); (**c**) before OPC; (**d**) after OPC.

Figure 11a shows the results based on the simulation, Figure 11b shows the local IDT diagram without correction, and Figure 11c shows the local IDT diagram with the best image correction.

As seen in the experimental SEM Figure 11c, the OPE causes the shape of the electrode and virtual electrode’s tail end to deviate from the design and carry a significant inward shrinkage. At the same time, it can be seen that the electrode and the virtual electrode’s tail end become significantly rounded. This deformation reduces the effectiveness of the virtual electrode and gap in suppressing transverse wave SAW leakage, which in turn reduces the quality factor (Q) of the SAW resonator. In addition, the end of the electrode and the dummy finger become sharp, and the tip discharge problem mentioned in the previous section may also occur, which reduces the reliability of the product operation, so it is necessary to adjust and optimize this problem.

From SEM Figure 11d, it can be observed that the tail-end deformation of the electrodes and virtual electrodes is significantly reduced, and the gap is reduced considerably after applying OPC, proving the feasibility of using OPC to the SAW filter design in this paper. Compared to Figure 11d, Figure 11c has a significant inward shrinkage in addition to a more rounded tail, resulting in a larger gap and reducing the filter’s ability to suppress the transverse wave SAW leakage. The corrected result Figure 11d shows that OPC improves the difference caused by OPE on the pattern of SAW resonators, basically eliminating the adverse effect caused by OPE, which will enhance the quality factor of the resonator, reduce the influence of external factor on the design effect, and improve the performance and stability of SAW filters.

The experimental results are consistent with the theoretical analysis. However, it can be seen from Figure 11d that there is still a slight rounding at the tail end of the electrode and the virtual electrode, which deviates slightly from the rectangle in the design graph. This is because the OPC of this experiment is a rule-based method using discrete correction parameters x1, x2, y1, and y2, so the rectangular parameters used in the OPC are only the optimal correction parameters within a specific range. To improve the correction effect, a more refined step or the application of the model method can be considered to find a way to eliminate the adverse effects caused by the OPE.

**Figure 12:**
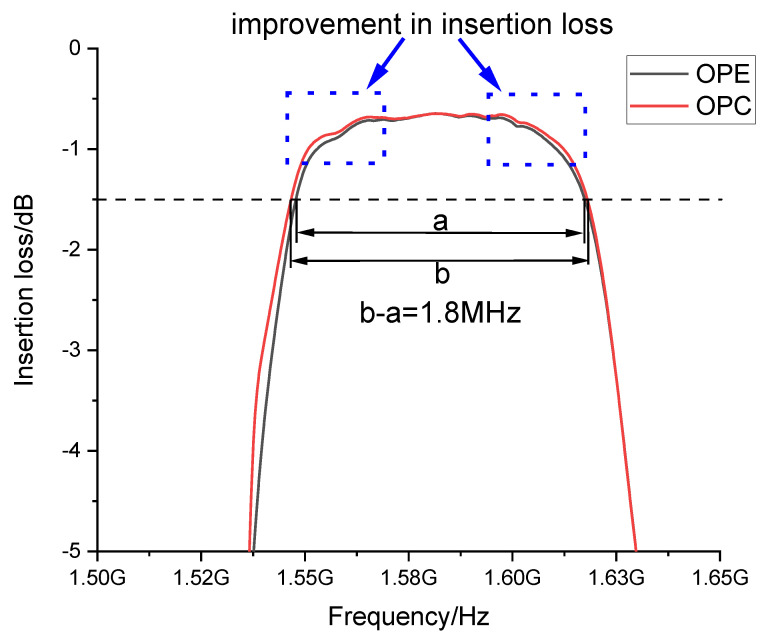
Insertion loss−frequency relationship before and after OPC.

Figure 12 shows the insertion loss curves of the SAW filter before and after OPC. Among them, the black curve represents the insertion loss−frequency relationship under the influence of OPE; while the red curve represents the insertion loss-frequency relationship after OPC. The insertion loss in the passband is reduced after correction compared to before correction, particularly in the blue dashed box, indicating that energy leakage is reduced, i.e., transmission efficiency is improved after correction. The bandwidth is increased by 1.8 MHz at −1.5 dB. The experimental results illustrate that the filter performance is effectively improved by using OPC. Therefore, OPC can effectively reduce the insertion loss of SAW filters and enhance the performance of SAW filters.

Based on the experimental results and the above analysis, a comparison of the OPC method with other methods is summarized in Table 3. All three methods in the table improve the resonator quality factor and reduce the filter’s insertion loss. Among them, the present method does not require additional cost for a slight change in electrode structure. Compared with the other two methods, the method proposed in this paper is less challenging to lithograph and does not require additional thin film layers, which is less costly.

## 5. Conclusions

In this paper, we use a rule-based OPC method to correct the rounding and indentation of the pattern due to the electrode gap and the narrow line width of the SAW resonator. The correction rectangles are selected by formulating suitable correction rules, and experiments are performed to verify the method. The experimental results confirm that OPC is capable to correct the distortion of the pattern, thus ensuring the ability of the virtual electrodes to suppress the lateral leakage of the SAW. As a result, the insertion loss of the SAW filter can be reduced, and the tip discharge problem can be avoided to improve the power handling of the filter.

## Figures and Tables

**Table 1:** Simulation parameter table.

Simulation Parameters	
Number of IDT	1 pair
Metallization ratio	0.5
IDT pitch	1.21 μm
Aperture	40 μm
Gap	0.125 λ/0.175 λ/0.25 λ
Dummy length	1.25 λ
Al	240 nm

**Table 2:** Measurement data when λ = 2.42 µm.

Lambda = 2.42 µm			
k	gap	h	a_max_
0	504 nm	1113 nm	605 nm
0.10	494 nm	998 nm	605 nm
0.15	470 nm	848 nm	605 nm
0.20	443 nm	550 nm	605 nm
0.25	419 nm	480 nm	615 nm
0.30	385 nm	455 nm	645 nm

**Table 3:** Comparison with other methods.

	Reference 3	Reference 2	The OPC Method Proposed in This Paper
Effect	Realized the purpose of reducing insertion loss and improving quality factor		
Method	Change of electrode structure	Laying Ta_2_O_5_ on the busbar, dummy electrode and electrode gap	Optical proximity effect correction
Pros and cons	1. Complicated IDT structure 2. Effective suppression of transverse SAW leakage	1. Complex process 2. High cost 3. Effective suppression of transverse SAW leakage	1. Weaken the influence of OPE 2. No additional cost is required 3. Effectively suppress transverse SAW leakage and avoid tip discharge

## Data Availability

Not applicable.
